# Supplementary material for: Epitaxy of wafer-scale single-crystal MoS2 monolayer via buffer layer control
Source: Nat Commun. 2024 Feb 28;15:1825. doi: 10.1038/s41467-024-46170-6 (PMC10901795; doi:10.1038/s41467-024-46170-6)
Supplement: Supplementary file 1 — Supplementary Information [file 41467_2024_46170_MOESM1_ESM.pdf]

Supplementary Information for
Epitaxy of wafer-scale single-crystal MoS_2 monolayer via buffer layer control

Lu Li[1,2], Qinqin Wang[1,2], Fanfan Wu[1,2], Qiaoling Xu[3,4], Jinpeng Tian[1,2], Zhiheng Huang[1,2], Qinghe Wang[5], Xuan Zhao[1,2], Qinghua Zhang[1,2], Qinkai Fan[1,2], Xiuzhen Li[1,2], Yalin Peng[1,2], Yangkun Zhang[1,2], Kunshan Ji[1,2], Aomiao Zhi[1,2], Huacong Sun[1,2], Mingtong Zhu[1,2], Jundong Zhu[1,2], Nianpeng Lu[1,2,3], Ying Lu[1,2], Shuopei Wang[3], Xuedong Bai[1,2,3], Yang Xu[1,2], Wei Yang[1,2], Na Li[3], Dongxia Shi[1,2,3], Lede Xian[3], Kaihui Liu[5], Luojun Du[1,2*] & Guangyu Zhang[1,2,3*]

[1]*Beijing National Laboratory for Condensed Matter Physics, Institute of Physics, Chinese Academy of Sciences, Beijing 100190, China*

[2]*School of Physical Sciences, University of Chinese Academy of Sciences, Beijing 100049, China*

[3]*Songshan Lake Materials Laboratory, Dongguan, Guangdong 523808, China*

[4]*College of Physics and Electronic Engineering, Center for Computational Sciences, Sichuan Normal University, Chengdu 610068, China*

[5]*Collaborative Innovation Center of Quantum Matter and School of Physics, Peking University, Beijing 100871, China*

Email: luojun.du@iphy.ac.cn; gyzhang@iphy.ac.cn

Supplementary Note 1. The unidirectional growth of MoS_2 domains

According to microscopy images over a ~1 mm^2 area (Supplementary Fig. 1), the degree of unidirectional alignment is larger than 99% for 4.5% MoO_3/S precursor ratio.

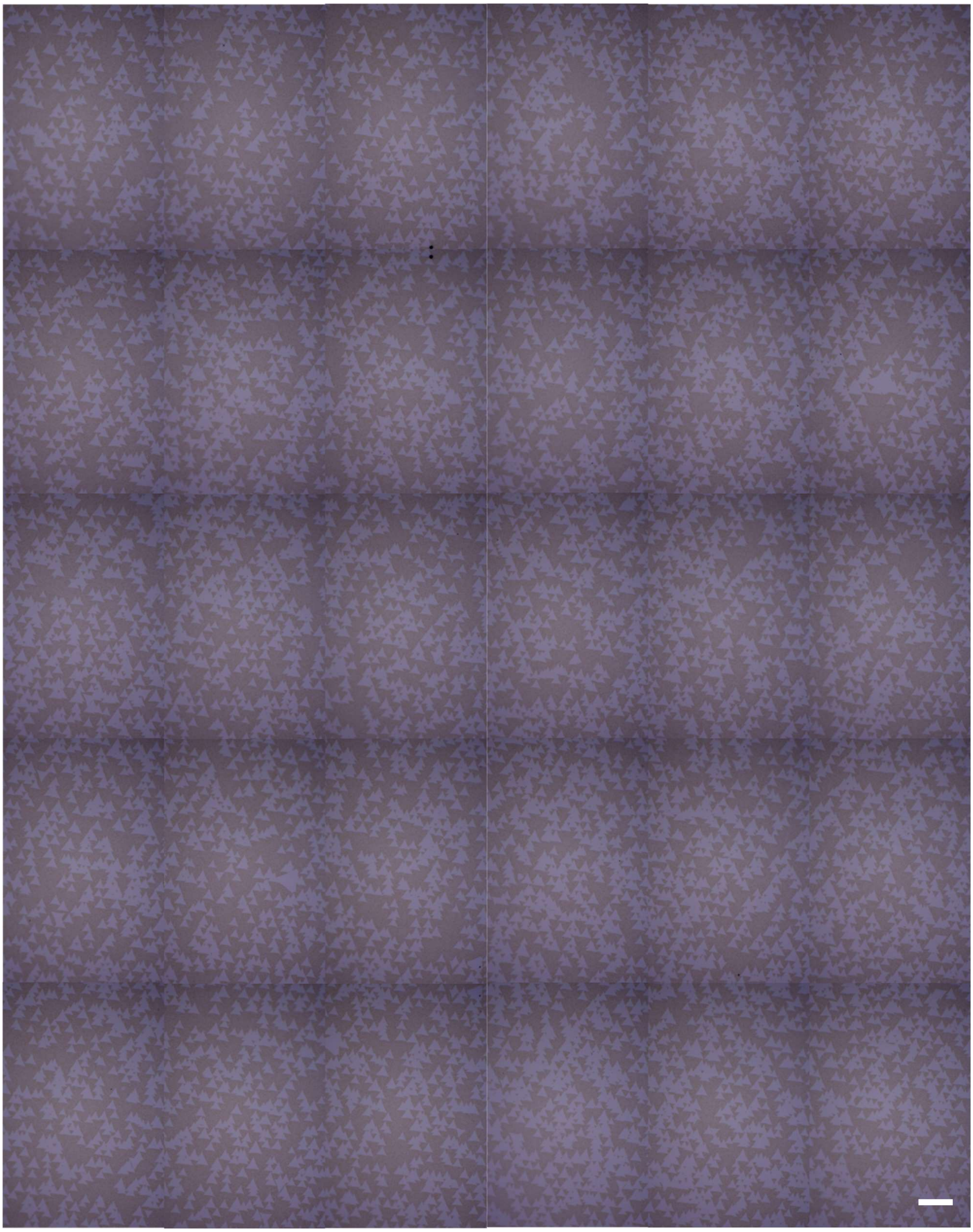

Supplementary Figure 1. The uniform and unidirectional growth of MoS_2 domains over a ~1 mm^2 area. Scale bars, 30 μm.

Supplementary Note 2. MoS_2 domains at different Mo/S precursor ratios

Supplementary Figure 2a presents the optical micrographs of the as-grown MoS_2 triangular domains under different MoO_3/S precursor ratios: 3.9%, 4.48%, 4.53%, 4.87%, 5% and 5.55%. The degree of unidirectional alignment can be continuously tuned from ~0 to ~100% by controlling the MoO_3/S precursor ratio (Supplementary Fig. 2b).

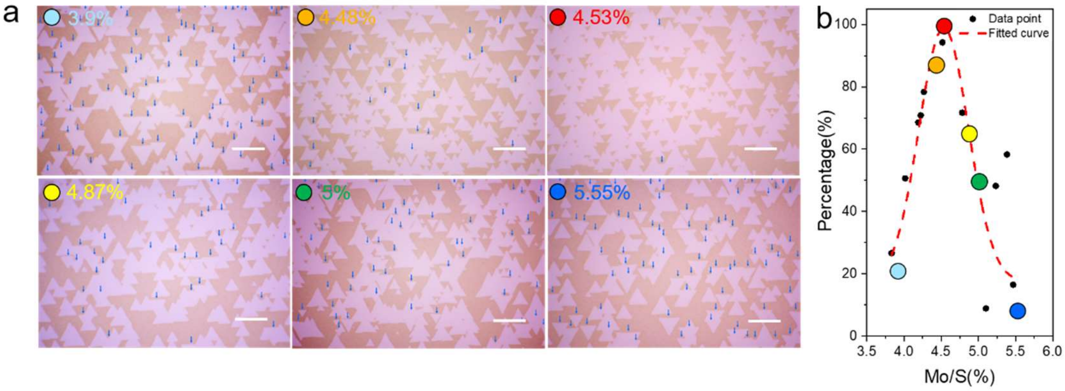

Supplementary Figure 2. (a) The optical microscopy images show MoS_2 domains at different Mo/S precursor ratios. Scale bar, 10 μm. (b) The degree of unidirectional alignment as a function of the MoO_3/S precursor ratio.

Supplementary Note 3. Unidirectional domain alignment on pure *c*-plane sapphires and other substrates.

In addition to *c*-plane sapphire substrates with a major miscut angle (~0.2°) towards M-axis in the main text, the unidirectional domain alignment has also been achieved by precisely controlling the S/MoO_3 precursor ratio on pure *c*-plane sapphires without an M-axis miscut (the degree of unidirectional alignment is ~99.2%, Supplementary Fig. 3a), *c*-plane sapphire substrates with different major miscut angles towards M-axis such as C/M-0.5° (the degree of unidirectional alignment is ~96.6%, Supplementary Fig. 3b) and C/M-3° (the degree of unidirectional alignment is ~99.5%, Supplementary Fig. 3c), *c*-plane sapphire substrates with a major miscut angle (~0.2°) towards other axes (the degree of unidirectional alignment is ~98.7% in Supplementary Fig. 3d and the degree of unidirectional alignment is ~96.4% in Supplementary Fig. 3e), and *c*-plane sapphire substrates with a major miscut angle (~0.2°) towards A-axis (the degree of unidirectional alignment is ~98.1%, Supplementary Fig. 3f). Therefore, we believe that our claim is valid for pure *c*-plane sapphires without an M-axis miscut and other substrates.

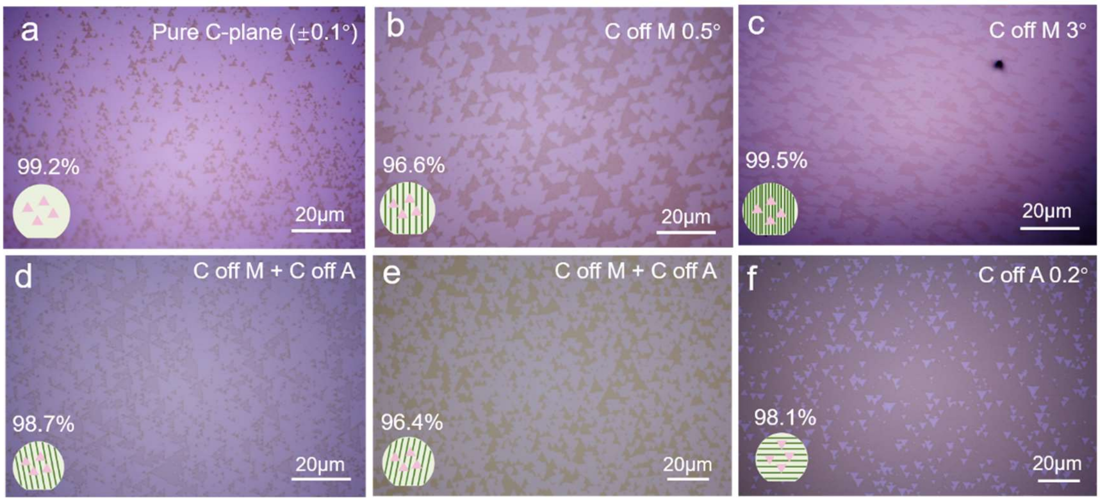

Supplementary Figure 3. Optical microscopy image of unidirectional MoS_2 domains on pure *c*-plane sapphires without an M-axis miscut (**a**), *c*-plane sapphire substrates with a 0.5° major miscut angles towards M-axis (**b**), a 3° major miscut angles towards M-axis (**c**), with a 0.2° major miscut angles towards other axes (**d**,**e**), and with a 0.2° major miscut angle towards A-axis (**f**).

Supplementary Note 4. The shape evolution of MoS_2 crystals

For our home-designed three-temperature-zone chemical vapor deposition (CVD) setup (Supplementary Fig. 27), there is a long distance between the substrate and MoO_3 source (>32 cm), as well as S source (>49 cm). Therefore, the gradient of both MoO_3 and S sources is very small and the MoS_2 crystals would maintain the same shape. Supplementary Figure 4 shows the optical microscopy images of CVD MoS_2 at different locations (~32-40 cm). Clearly, the MoS_2 crystals keep the triangular shape. Furthermore, for all the MoO_3/S ratios we have investigated (ranging from ~3.83% to ~5.55%), the concentration of S source is always larger than that of MoO_3 source. Consequently, the shape of MoS_2 crystals would keep unchanged, as in our experimental results (Supplementary Fig. 4)

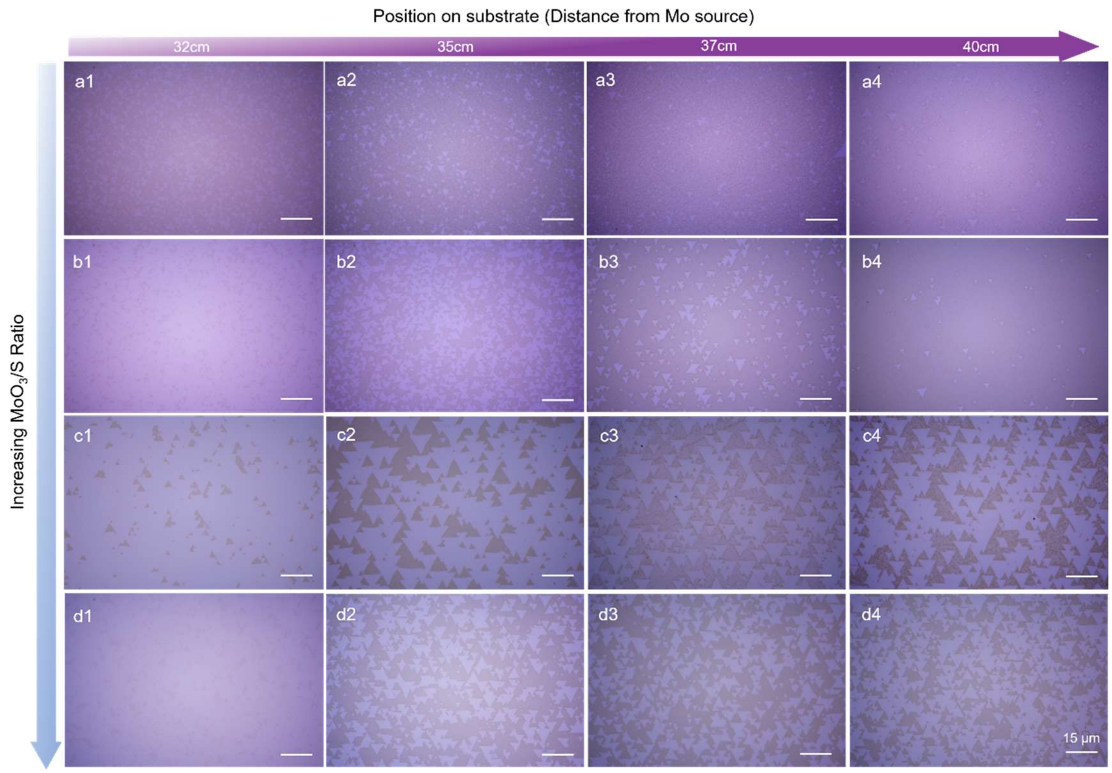

Supplementary Figure 4. Representative optical images of MoS_2 crystals grown at different MoO_3/S (a)-(d) at several different positions on the substrate (1)-(4).

Supplementary Note 5. The AFM image of MoS_2 film

A scratch was made on a continuous MoS_2 film to measure the height of the MoS_2, and the AFM image is shown in Supplementary Fig. 5a. The height results from the dashed line (Supplementary Fig. 5b) show that MoS_2 is about 0.7 nm, indicating the monolayer nature.

The AFM images taken from different locations across a 2-inch wafer (Supplementary Fig. 6) manifest a uniform and wrinkle-free monolayer MoS_2 film.

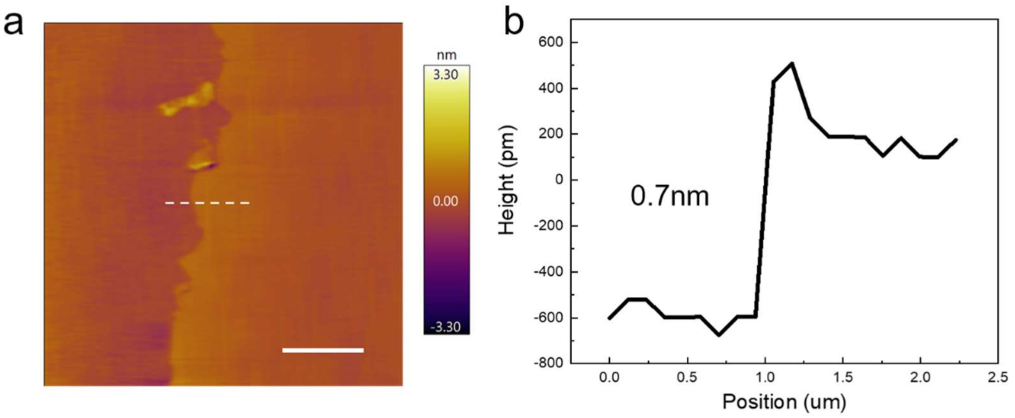

Supplementary Figure 5. (a) The AFM image of the scratched edge of MoS_2. Scale bar, 2 μm. (b) The height results from the dashed line.

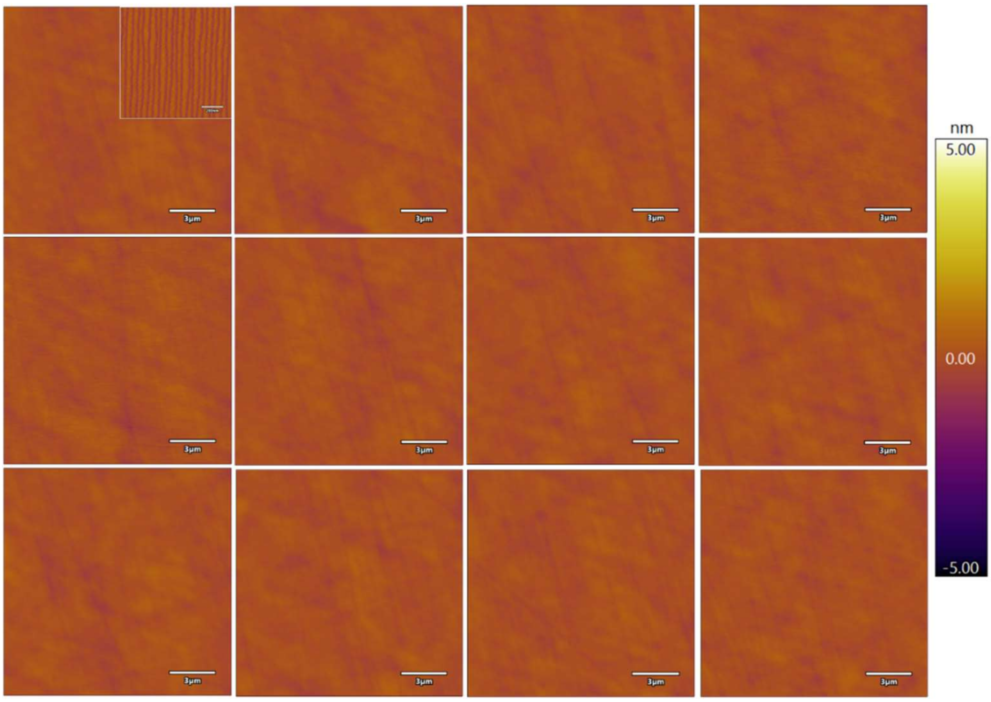

Supplementary Figure 6. Typical AFM images of MoS_2 grown on c-Al_2O_3 at different areas.

Supplementary Note 6. General *c*-plane sapphire substrates with mono-step

In a recent work[1], the bi-steps and thus single-type surface symmetry are formed by annealing at high temperatures, which enable the unidirectional alignment. However, in our studies, the used *c*-plane sapphire substrates are the general substrate with mono-step, rather than bi-steps.

1) Supplementary Figure 7a shows the XRD rocking curve of our *c*-plane sapphire substrates with unidirectional MoS_2 on it, demonstrating a major miscut angle towards M axis of 0.135°, which agrees well with the parameters provided by the supplier of HeFei crystal Technical Material Co., Ltd. (*c*-plane sapphire with a major miscut angle towards M axis of ~ 0.2 ± 0.1°).

2) Supplementary Figures 7b and 7c shows the AFM image of our *c*-plane sapphire substrates, indicating mono-step height H of ~ 0.16 nm and terrace width W of ~ 66.5 nm. The ratio between step height H and terrace width W, i.e., $H/W = 0.16nm/66.5nm = 0.0024$, accordance with the XRD data ($tan\ 0.135° = 0.002356$).

3) Our *c*-plane sapphire substrates with a major miscut angle towards M axis of ~ 0.2° have mono-step are quite reasonable because our substrates are annealing at 980°C, much lower than the required temperature (>1400 °C) to form bi-steps[1].

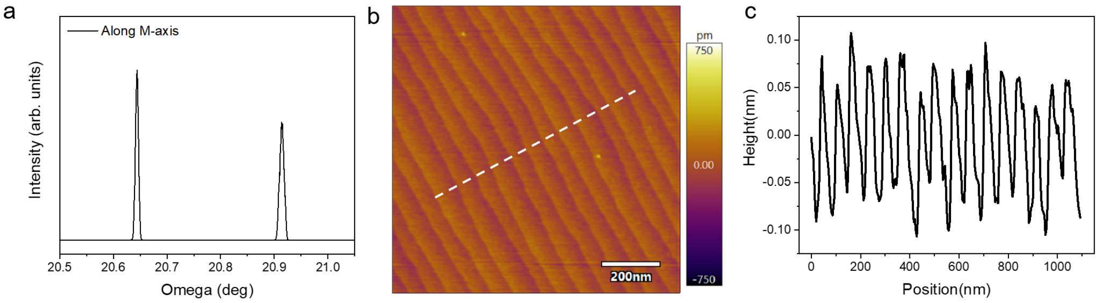

Supplementary Figure 7. (**a**) XRD rocking curve of *c*-plane sapphire substrates with unidirectional MoS_2 on it, demonstrating a major miscut angle towards M axis of 0.135°. (**b,c**) AFM characterization of sapphire substrates, indicating surface step height of ~0.16 nm and terrace width of ~66.5 nm.

Supplementary Note 7. The epitaxial relationship of MoS_2 on c-plane sapphire

According to the AFM images (Supplementary Fig. 8), the MoS_2 <11$\bar{2}$0> direction (MoS_2 zigzag) is parallel to the <10$\bar{1}$0> direction of sapphire substrate (M axis), and the MoS_2 <10$\bar{1}$0> direction (MoS_2 armchair) is parallel to the sapphire <11$\bar{2}$0> direction (A axis).

We performed transmission electron microscopy (TEM) characterization at the atomic scale and confirmed that the triangular MoS_2 domains have zigzag edges (Supplementary Fig. 9).

The epitaxial relationship of MoS_2 on c-plane sapphire is further confirmed by in-plane X-ray diffraction (XRD) measurements. In the XRD θ-2θ scan, only the diffraction peak of MoS_2 (10$\bar{1}$0) plane and α-Al_2O_3 (11$\bar{2}$0) plane are detected simultaneously in the single oriented MoS_2 film at the same φ angle (red line, Supplementary Fig. 10a). Furthermore, the XRD φ scan (Supplementary Fig. 10b) show that the peaks of MoS_2 (10$\bar{1}$0) plane coincide with that of α-Al_2O_3 (11$\bar{2}$0) plane, indicate the single epitaxial relationship of MoS_2 (10$\bar{1}$0) // α-Al_2O_3 (11$\bar{2}$0).

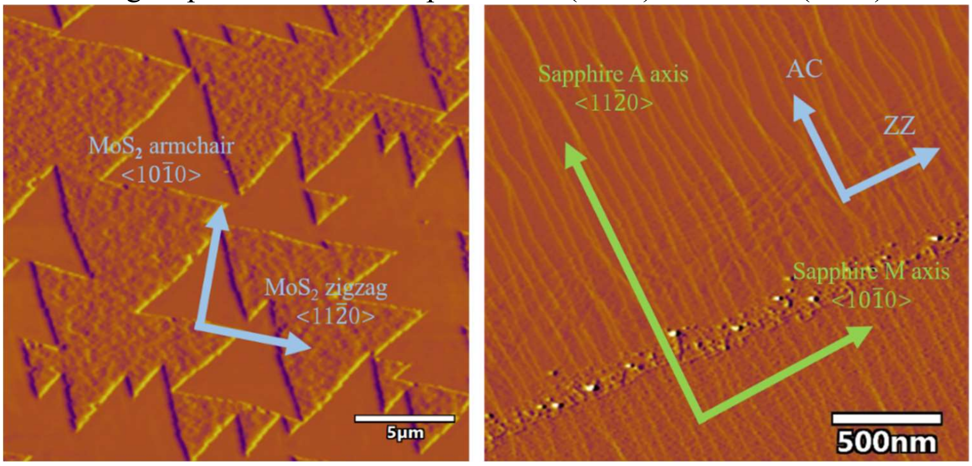

Supplementary Figure 8. The AFM images of triangular domains show that the zigzag <11$\bar{2}$0> direction of single oriented MoS_2 is parallel to the <10$\bar{1}$0> direction of sapphire substrate, and the armchair <10$\bar{1}$0> direction of MoS_2 is parallel to the sapphire step <11$\bar{2}$0> direction.

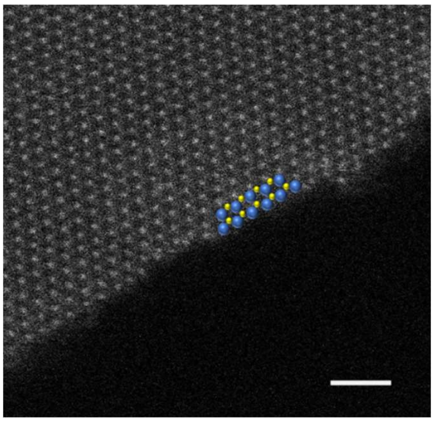

Supplementary Figure 9. STEM images show the Mo-Zigzag edges of MoS_2 domains. Scale bars, 1 nm.

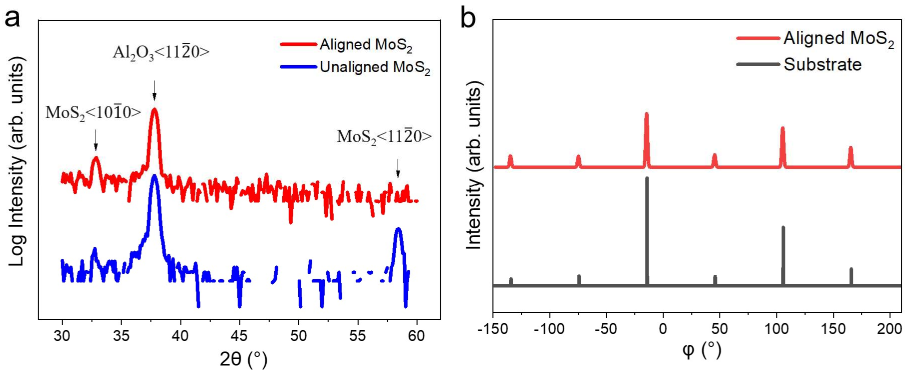

Supplementary Figure 10. (a) In-plane XRD θ–2θ diffractogram. (b) In-plane XRD φ scan of the $(10\bar{1}0)$ planes of MoS_2 and the $(11\bar{2}0)$ planes of α-Al_2O_3.

Supplementary Note 8. The mechanism of unidirectional MoS_2 growth

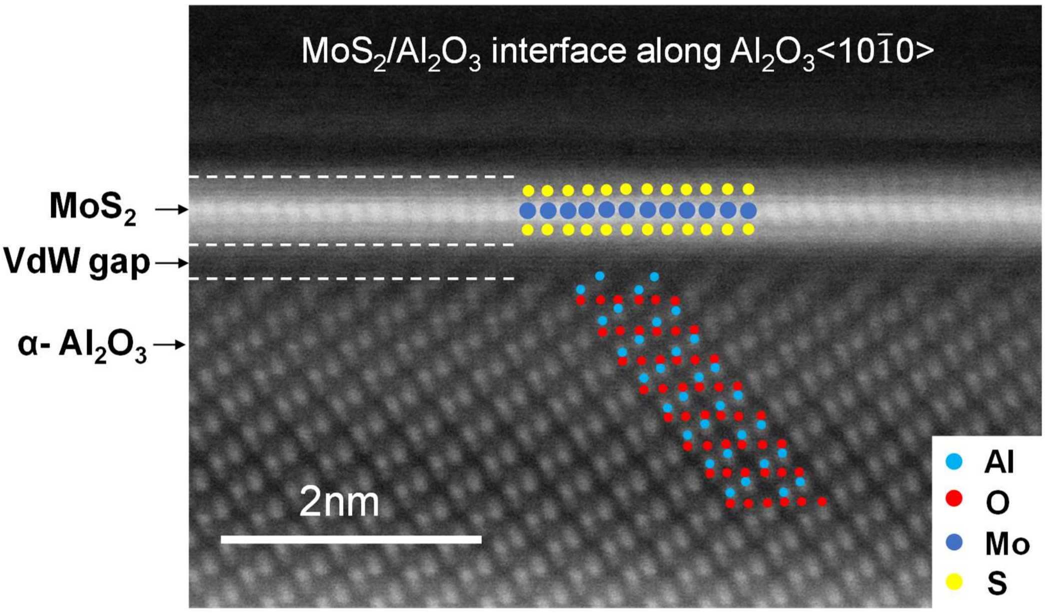

Supplementary Figure 11. Cross-sectional HAADF-STEM images of a MoS_2 grown on the α-Al_2O_3 (0001) substrate along the α-Al_2O_3 <$10\bar{1}0$> direction, where the degree of unidirectional alignment is ~ 0.

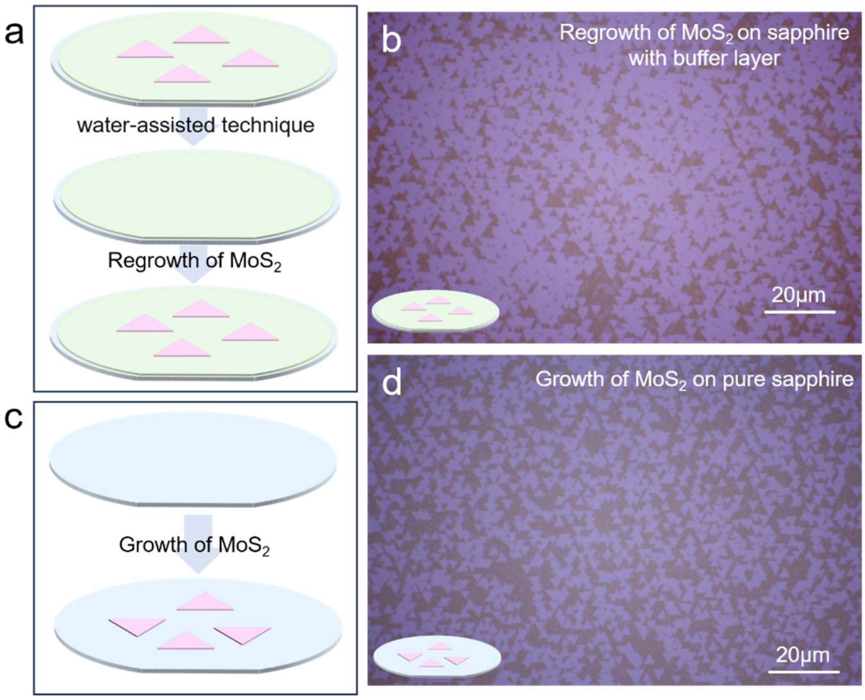

Supplementary Figure 12. (**a**) Schematic illustration of removal and regrowth of unidirectional MoS_2. The as-grown unidirectional MoS_2 is removed from *c*-plane sapphire substrate by water-assisted transfer method, which would perfectly maintain the formed buffer layer. (**b**) Optical micrographs of the regrown MoS_2 with unidirectional domain alignment. (**c,d**) Illustration and optical micrographs of MoS_2 domains grown on an unused new sapphire substrate, showing two antiparallel domains.

Supplementary Note 9. The structure of the buffer layer

Supplementary Figure 13 shows the high-resolution XPS spectrum of Mo 3*d*, after removing the as-grown unidirectional MoS_2 by a non-destructive water-assisted technique. A deconvolution and curve fitting reveal that the Mo 3*d* spectrum can be decomposed into a doublet corresponding to $3d_{5/2}$ Mo^{5+} ($3d_{3/2}$ Mo^{5+}).

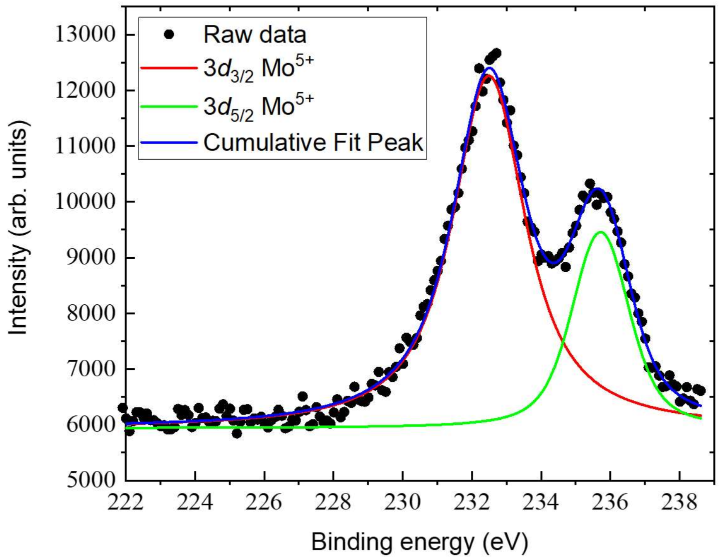

Supplementary Figure 13. The high-resolution XPS spectrum of Mo 3*d*.

Based on the XPS results and previous results of MoS_2 epitaxy on β-Ga_2O_3 (001)[2], we infer that one possible configuration of the buffer layer is O−Mo−O−Al, with Mo exhibiting a (+5) oxidation state. To determine the detailed structure of the O−Mo−O−Al buffer layer, we perform density functional theory (DFT) calculations with the Vienna ab initio software package[3]. The Perdew-Burke-Ernzerhof (PBE) exchange-correlation functionals are employed[4], along with DFT-D2 method of Grimme van der Waals corrections[5]. An energy cutoff of 520 eV for the plane wave basis sets and a Γ-centered k-mesh of 1×1×1 is used for geometry optimization and electronic structure calculations. The convergence of total energy with respect to these parameters is examined and found to reach the level of less than 0.001 eV/f.u. To prevent artificial interactions between periodic slab images, a vacuum thickness greater than 15 Å was applied. The Al_2O_3 (001) surfaces are modeled using large supercell slabs containing 9 atomic layers. The bottom 5 layers of atoms are kept fixed at their optimized bulk positions and other atomic positions are fully optimized with the lattice constant for the supercell kept fixed at a value corresponding to the experimental lattice constant for a 1×1 unit cell.

Supplementary Figure 14 shows the calculated the structure of the O−Mo−O−Al buffer layer on Al_2O_3 surface after fully structural relaxation. Generally, every O atom under the Mo layer is connected to two Al and two Mo atoms, and every Mo atom is connected to 3 O atoms under it. Meanwhile, every O atom above the Mo layer is connected to two Mo atoms and 3 O atoms are assigned to 1 Mo atom. Further, MoS_2 triangles with different orientations are positioned on the surface of the Al_2O_3 substrate with O−Mo−O−Al buffer layer. The DFT results reveal that the 60° structure, compared to the 0° structure, has a lower free energy of 110 meV, demonstrating a preferred growth orientation for MoS_2 triangles.

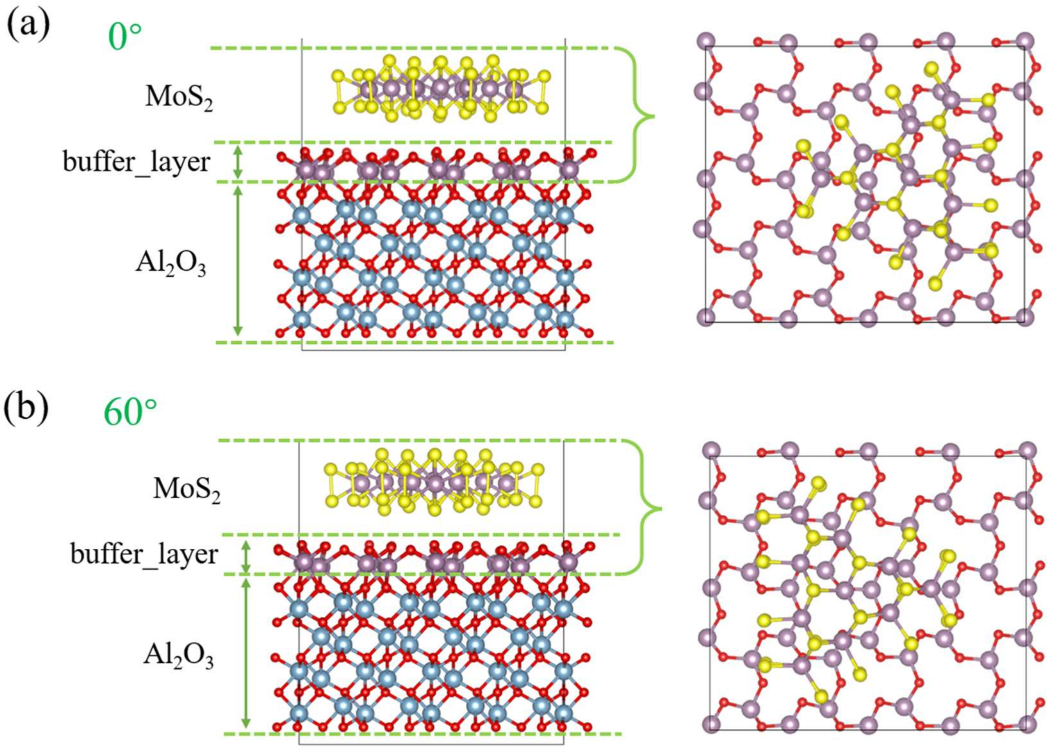

Supplementary Figure 14. The atomic structures of MoS_2 growth on α-Al_2O_3 (001) with buffer layer. The side view of the relaxed MoS_2 layer in 0° (a) and 60° (b) directions grown on Mo^{+5}/Al_2O_3 substrate and the corresponding top view of MoS_2 on the buffer layer. The Mo, S, O and Al atoms are shown in purple, yellow, red, and blue respectively.

Supplementary Note 10. The atomic-resolution HAADF-STEM images of MoS_2 samples

Supplementary Figure 15 show more atomic-resolution HAADF-STEM images from different merging areas, the atomic-resolution HAADF-STEM images are filtered to enhance the contrast and no stitched grain boundary is found.

The Vs were denoted in the yellow circles in Supplementary Figure 16, and the average concentration of sulfur vacancy is about $\sim 5.2 \times 10^{12}\ cm^2$. which is an order of magnitude lower than that of previously reported exfoliated MoS_2.

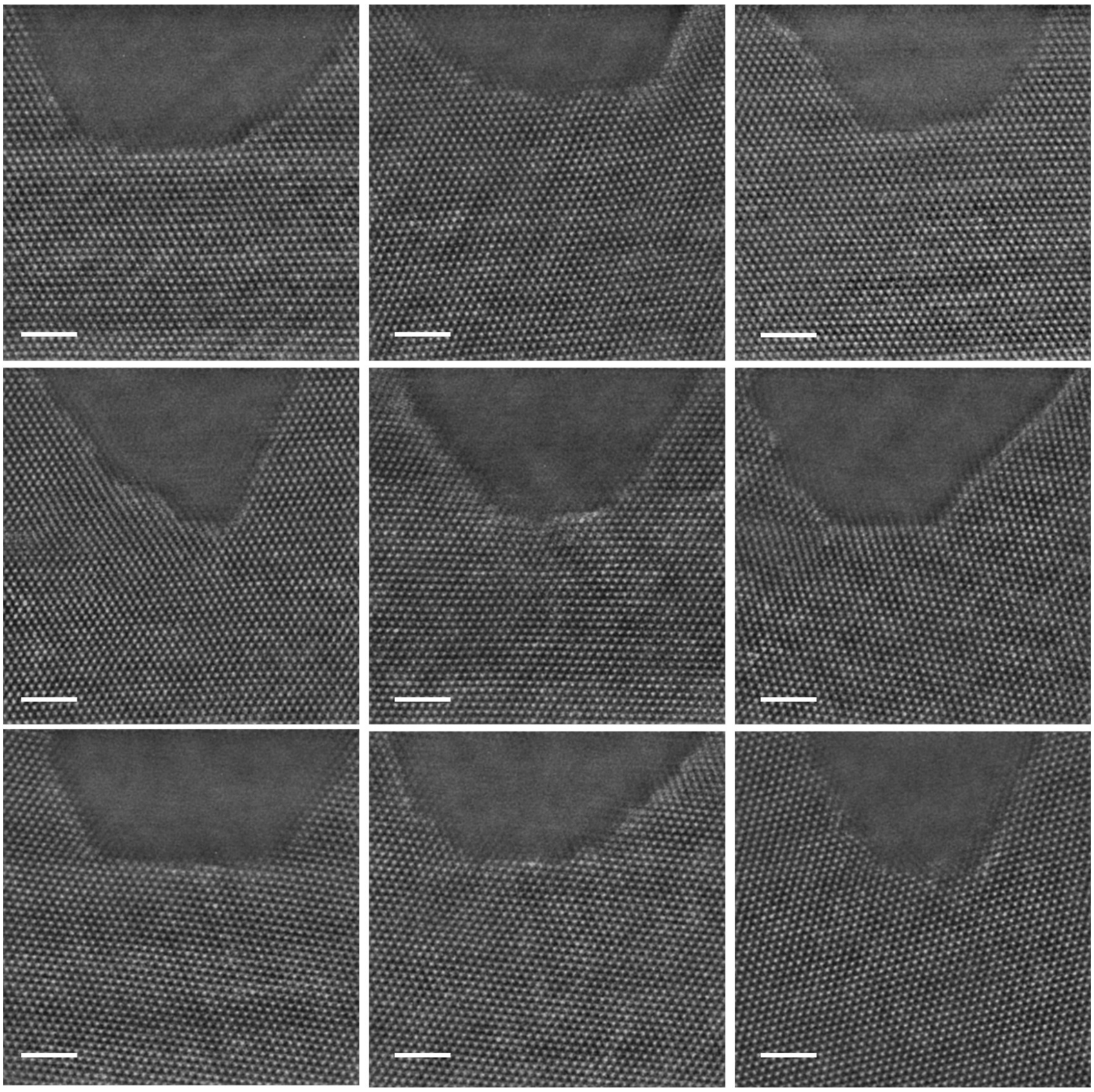

Supplementary Figure 15. HAADF-STEM images of the merging areas between neighboring unidirectional domains. Scale bar, 2 nm.

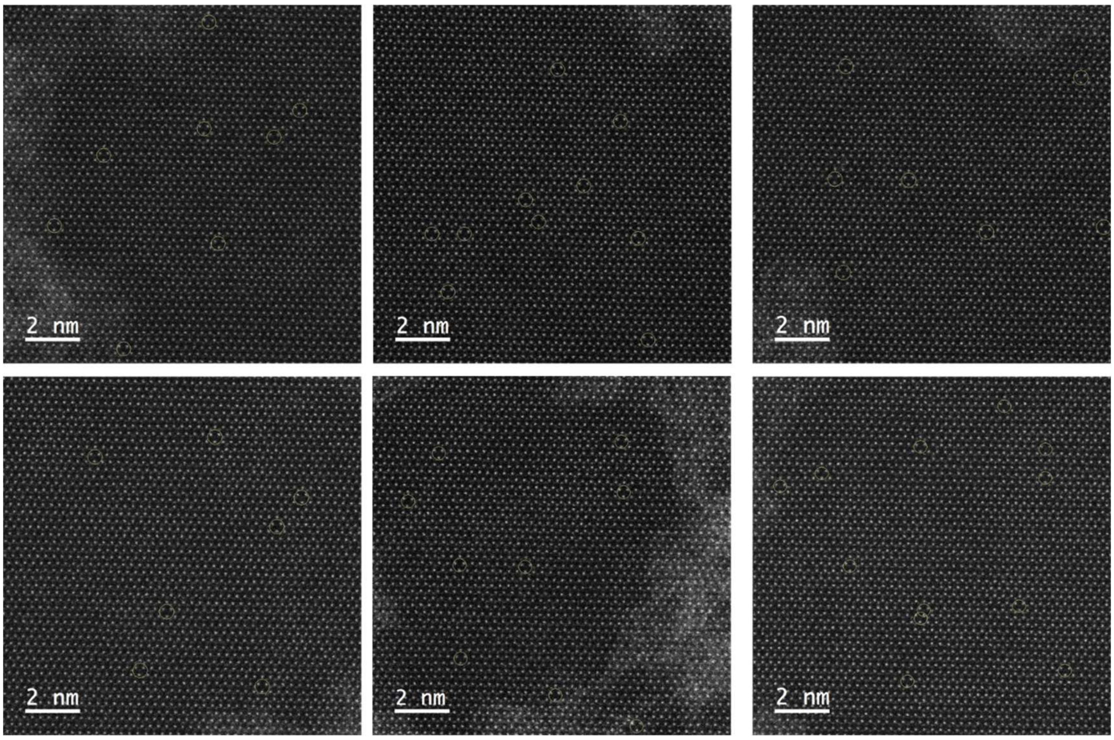

Supplementary Figure 16. The atomic-resolution STEM images of MoS_2 samples.

Supplementary Note 11. Etching results verify seamless stitching

The samples are heated at 150°C for 30 minutes under moisture-rich ambient conditions for thermal oxidation. No boundaries are observed for single oriented MoS_2 films (Supplementary Fig.17a), but boundaries can be clearly seen in multiple oriented MoS_2 films under the same condition (Supplementary Fig. 17b).

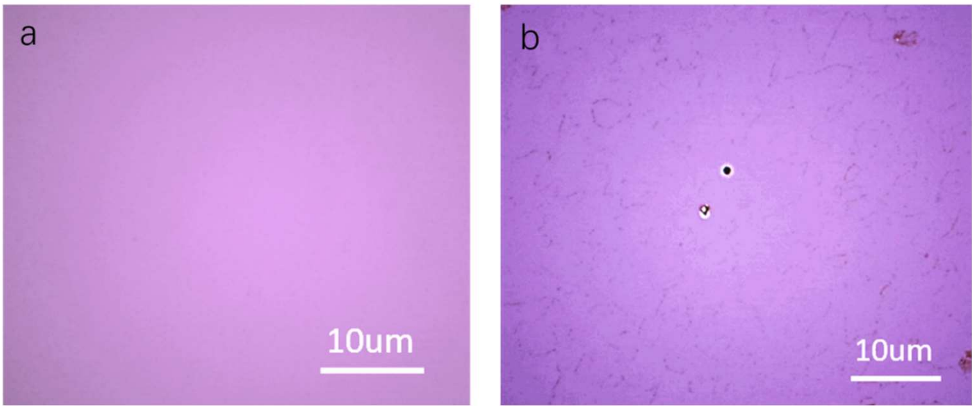

Supplementary Figure 17. Optical images of single oriented MoS_2 films (a) and multiple oriented MoS_2 films (b) after hot H_2O vapour etching.

Supplementary Note 12. The LEED patterns of MoS_2 films

Due to the three rotational symmetry of MoS_2, the intensity of three diffraction points in LEED diffraction are much higher than those of the other three points, indicating that the obtained MoS_2 films have a single domain orientation (Supplementary Fig. 18a). By contrast, the intensities of the six diffraction points in

LEED diffraction are more uniform for continuous films with a mixture of two orientation domains (Supplementary Fig. 18b).

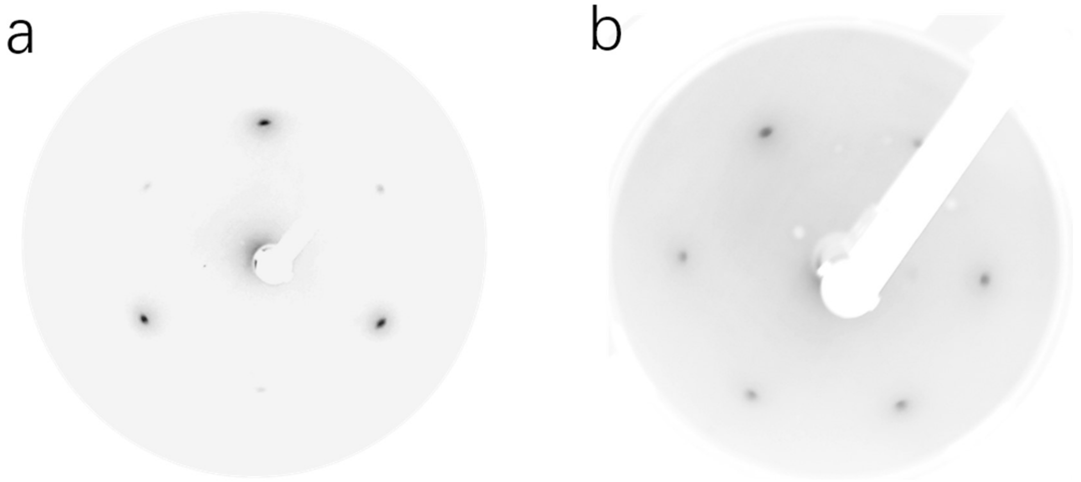

Supplementary Figure S18. The LEED patterns of single oriented MoS_2 films taken at the electron energy of 95eV (a) and the LEED patterns of multiple oriented MoS_2 films taken at the electron energy of 85eV (b).

Supplementary Note 13. High resolution PL and Raman mapping of MoS_2 single-crystal films

We perform high resolution PL and Raman mapping on MoS_2 wafers. Supplementary Figure 19 shows the PL and Raman mapping of MoS_2 single-crystal films: A_{1g} intensity (Supplementary Fig. 19a), E^{1}_{2g} intensity (Supplementary Fig. 19b) and the peak difference △ between E^{1}_{2g} and A_{1g} (Supplementary Fig. 19c), PL peak intensity (Supplementary Fig. 19d), PL peak position (Supplementary Fig. 19e) and FWHM (Supplementary Fig. 19f). According to the statistics analysis, the average peak distance between the Raman peaks E^{1}_{2g} and A_{1g} of MoS_2 is about 20.5 ± 0.5 cm^{-1}, and the average PL peak position is 1.86 eV ± 3 meV, and the average PL peak width at half height is 77 meV ± 2 meV. No apparent changes in the peak position and linewidth of both phonons and excitons are observed, illustrating the wafer-scale uniformity.

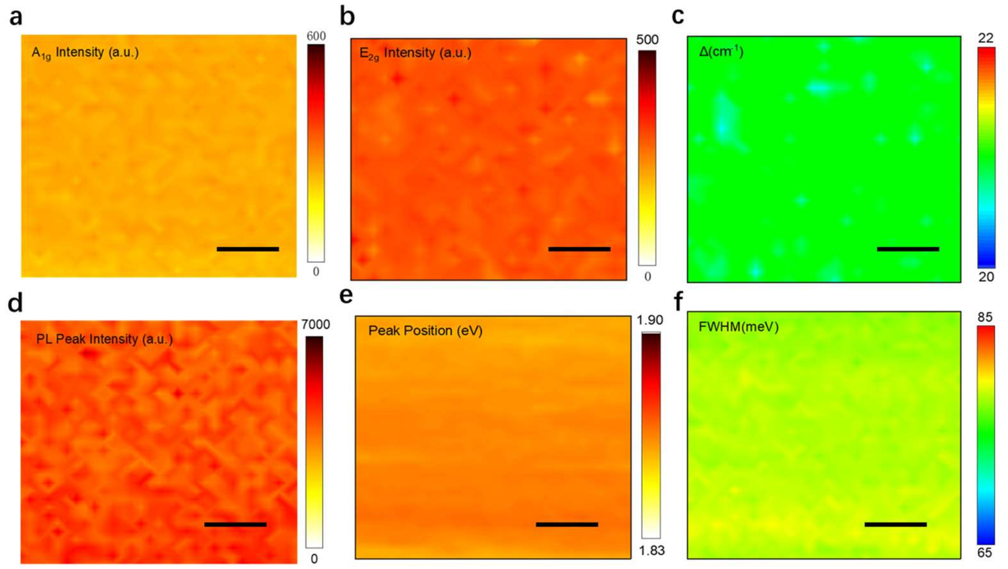

Supplementary Figure 19. Raman and PL mapping of MoS_2 single-crystal films. Raman spectra mapping in terms of A_{1g} intensity (a), E^1_{2g} intensity (b) and the peak difference $\triangle$ between E^1_{2g} and A_{1g} (c). (d,e,f). PL mapping of peak intensity (d), peak position (e) and FWHM (f), respectively. Scale bars, 5 μm.

Supplementary Note 14. Optical micrographs of single-crystal MoS_2 at different areas

According to microscopy images taken at different locations across a 2-inch range (Supplementary Fig. 20), the excellent wafer-scale uniformity of the as-grown monolayer MoS_2 single crystal is scrutinized.

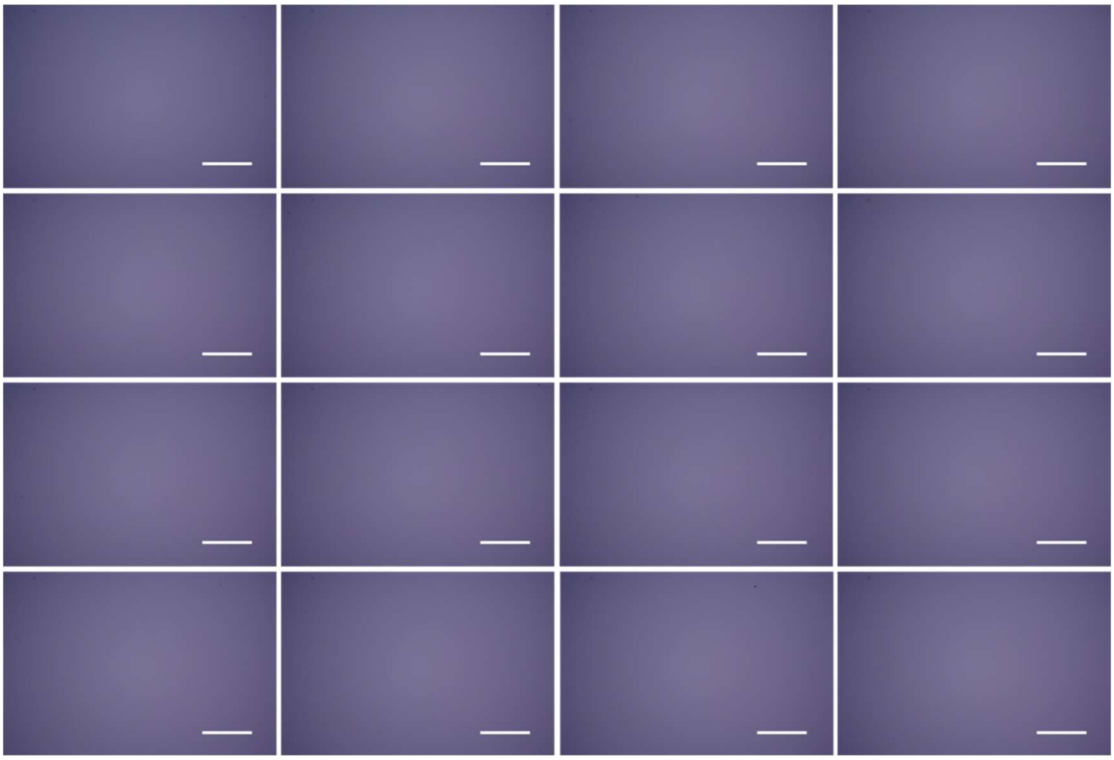

Supplementary Figure 20. Typical optical micrographs of MoS_2 grown on c-Al_2O_3 at different areas. Scale bar, 30 μm.

Supplementary Note 15. PL spectra of exfoliated monolayer MoS_2

Non-polarized PL spectra of exfoliated monolayer MoS_2 were performed and the full width at half maximum of the exfoliated monolayer MoS_2 is ~115 meV at 298K (Supplementary Fig. 21a) and ~53 meV at 10K (Supplementary Fig. 21b), which is about double of the values in our epitaxial monolayer MoS_2. The helicity-resolved PL spectra of the exfoliated monolayer MoS_2 was also performed at 10 K, excited by σ^+ radiation on resonance with the A exciton at 633 nm (1.96 eV) (Supplementary Fig. 21c), and the degree of exciton valley polarization is about 50% (Supplementary Fig. 21d).

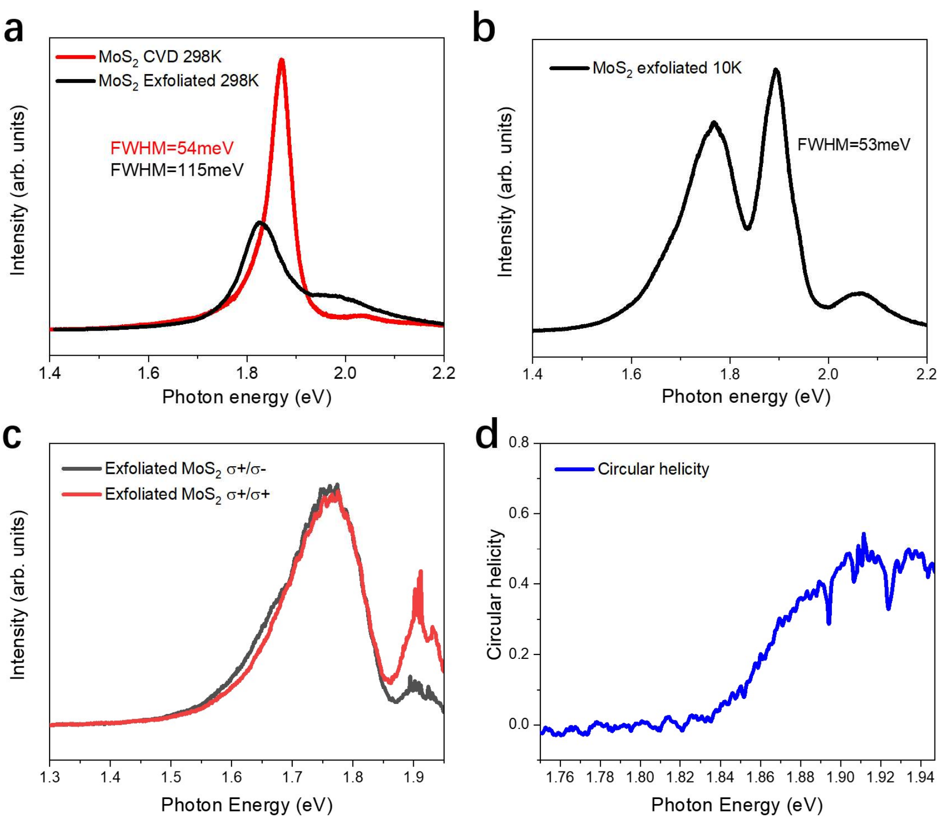

Supplementary Figure 21. Optical characterization of exfoliated MoS_2. (a) Typical room-temperature PL spectra of exfoliated MoS_2 and CVD-grown MoS_2. (b) Typical PL spectra of exfoliated MoS_2 at 10K. (c) Circularly polarized photoluminescence spectra of exfoliated MoS_2 at 10K. (d) Circular polarization calculated from the PL spectra in c.

Supplementary Note 16. The transfer characteristics of h-BN encapsulated devices

Supplementary Figure 22a (22c) shows the atomic force microscopy (AFM) image of the top (bottom) *h*-BN layer for *h*-BN encapsulated device in the main text. The corresponding height profiles (Supplementary Figs. 22b and 22d) indicate that the thickness of top and bottom *h*-BN layers is ~27 nm and ~34 nm, respectively.

Supplementary Figures 23a and 23b show a metal/*h*-BN/metal structure for capacitance measurement. The effective area between top and bottom metal electrodes is 20×33+5×3 μm^2; the thickness of *h*-BN is ~54 nm (Supplementary Fig. 23c). The measured capacitance is $\sim 382 \times 10^{-15} F$. Then we can extract the dielectric constant of *h*-BN $\varepsilon_r = \frac{C \cdot d}{\varepsilon_0 \cdot S} = \frac{382 \times 10^{-15} F \times 54 nm}{8.854 \times 10^{-12} F/m \times (20 \times 33 + 5 \times 3) \mu m^2} = 3.452$, which is in good agree with previous results[6,7]. Based on the measured dielectric constant of *h*-BN, we can obtain the capacitance per unit area for top gate ($C_i = \frac{\varepsilon 0 \cdot \varepsilon r}{d} = \frac{8.85 \times 10^{-12} F/m \times 3.45}{27 \times 10^{-9} m} = 1.131 \times 10^{-7}$ F/cm^2) and bottom gate ($C_i = \frac{8.85 \times 10^{-12} F/m \times 3.45}{34 \times 10^{-9} m} = 0.872 \times 10^{-7}$ F/cm^2).

The *h*-BN encapsulated devices with different channel length fabricated utilizing the pick-up technique (see method). Photograph of the *h*-BN encapsulated devices is

shown in Supplementary Fig. 24a. Supplementary Figures 24b and c show the room-temperature output (Supplementary Fig. 24a) and transfer (Supplementary Fig. 24a) characteristics of a *h*-BN encapsulated device with channel length of 2.7 μm and channel width of 1 μm. We calculated the variation of device mobility with top gate voltage for different channel lengths (Supplementary Fig. 24d), and the extracted field-effect mobility can reach ~ 140 $cm^2s^{-1}V^{-1}$. Supplementary Figure 24e-l show the transfer curves of MoS_2 device with different channel length: 5 μm (Supplementary Fig. 24e), 6.3 μm (Supplementary Fig. 24f), 8.6 μm (Supplementary Fig. 24g), 11.1 μm (Supplementary Fig. 21h), 14.2 μm (Supplementary Fig. 24i), 6.7 μm (Supplementary Fig. 24j), 27.5 μm (Supplementary Fig. 24k), 30.9 μm (Supplementary Fig. 24l).

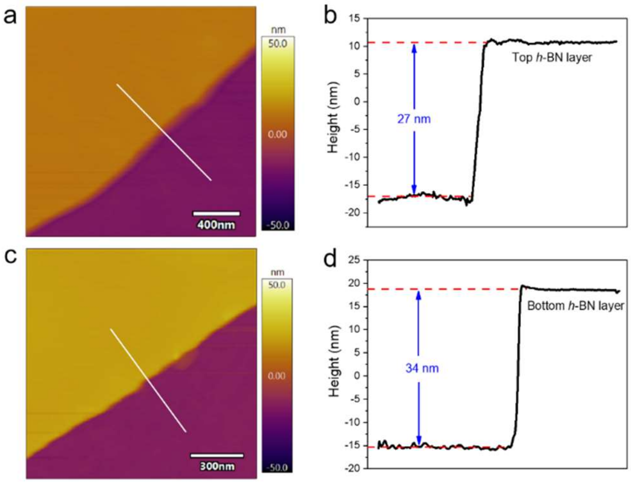

Supplementary Figure 22. (a,b) The AFM image of the top *h*-BN layer (a) and its corresponding height profiles (b). (c,d) The AFM image of the bottom *h*-BN layer (c) and its corresponding height profiles (d).

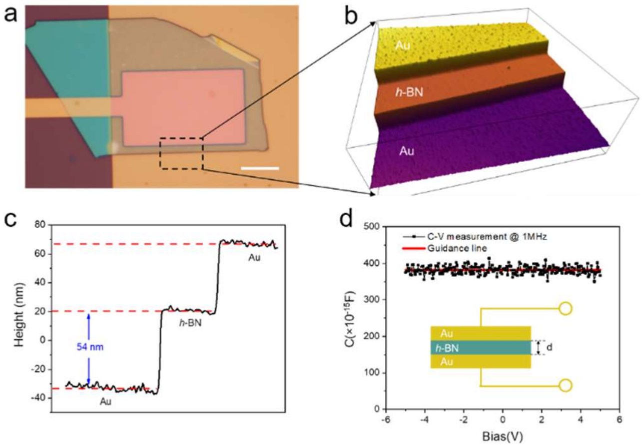

Supplementary Figure 23. (a) Optical image of a metal/*h*-BN/metal structure. The effective area between top and bottom metal electrodes is 20×33+5×3 μm^2. (b,c) The thickness of *h*-BN is ~54 nm measured by AFM. (d) C-V curves. The measured capacity is 382 fF.

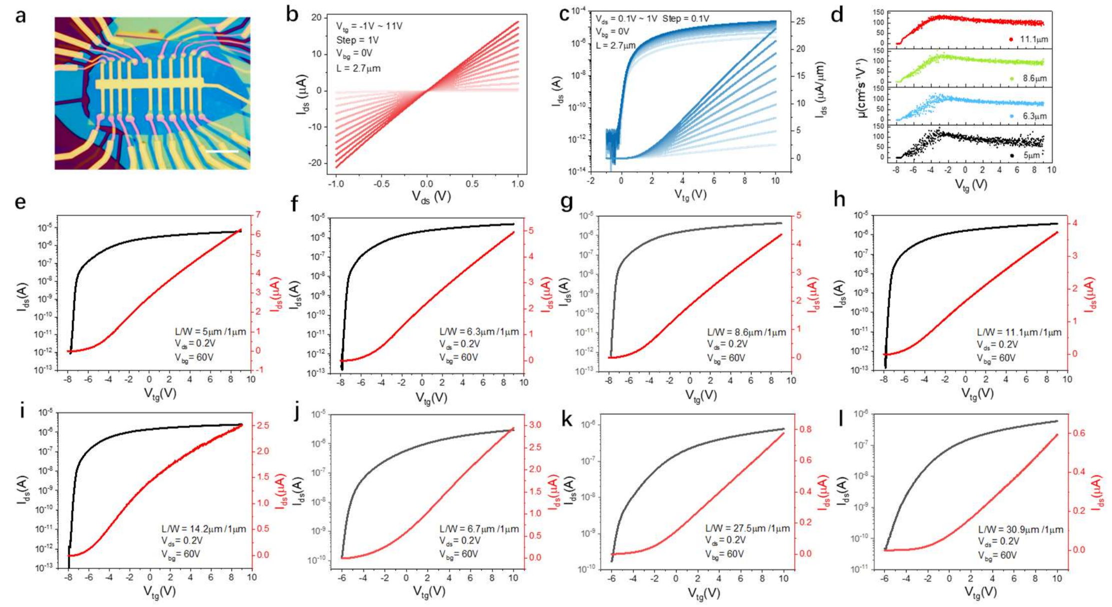

Supplementary Figure 24. FET performance of single MoS_2 devices. (a). Photograph of the single MoS_2 device. Scale bar, 10 μm. (b,c) Output and transfer curves of a *h*-BN encapsulated MoS_2 device with channel length/width (L/W) of 2.7/1 μm. V_{bg} = 0V. (d) The mobility with the change of V_{tg} at different channel lengths, W = 1μm, V_{bg} = 60V, V_{ds} = 0.2V. (e-l) Transfer curves of a *h*-BN encapsulated MoS_2 device with different channel.

Supplementary Note 17. Batch produced uncapsulated MoS_2 devices performance

Batch production of uncapsulated FET arrays over several centimeters is shown in Supplementary Fig. 25a. The 100 random FETs show an average on/off ratio ~10^8 (Supplementary Fig. 25c) and the statistical distribution of device mobility is shown in Supplementary Fig. 25f. Supplementary Figure 25d and 25e show the output (Supplementary Fig. 25d) and transfer (Supplementary Fig. 25e) curves of a typical device with channel length/width of 5/40 μm.

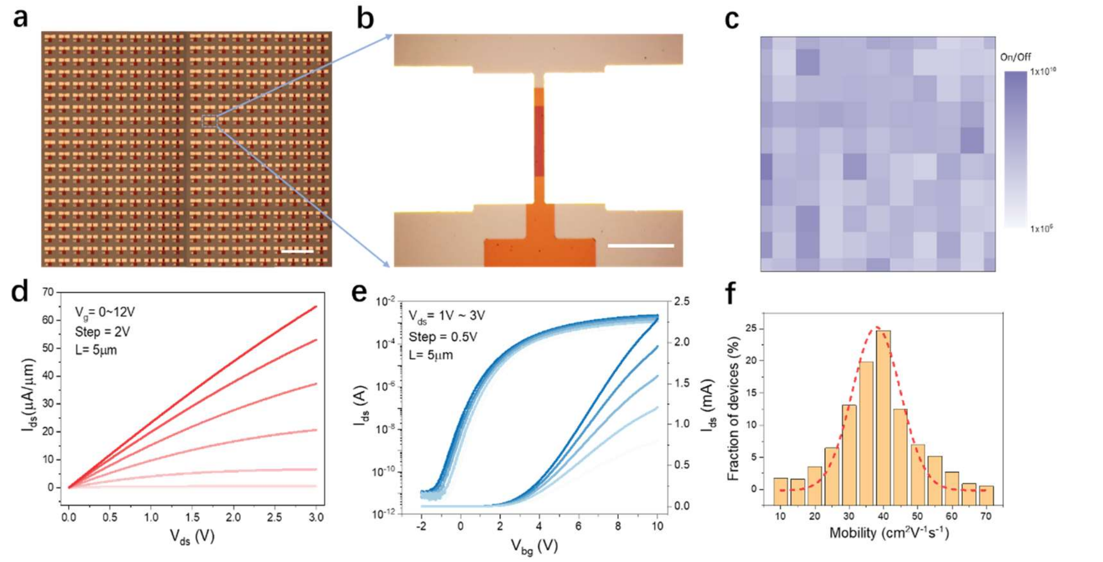

Supplementary Figure 25. FET performance of batch produced MoS_2 devices. (a) Photograph of a fabricated FET array. Scale bar,1 mm. (b) Enlarged image of a unit cell, Scale bar,45 μm. (c)

The on/off ratio of 100 random FETs. (d) Output and (e) transfer curves of a typical device with channel length/width of 5/40 μm. (f) The statistical distribution of device mobility.

For our short-channel HfO_2 devices in the main text, the corresponding C-V curve with the measurement frequency of 1 kHz, and amplitude of 50 mV is shown Supplementary Fig. 26. The capacitance per unit area at V=4 V is $C_i = \frac{1.15\times10^{-1}\ F}{100\times10^{-6}\times100\times10^{-6}m} = 1.15\times10^{-6}\ F/cm^2$.

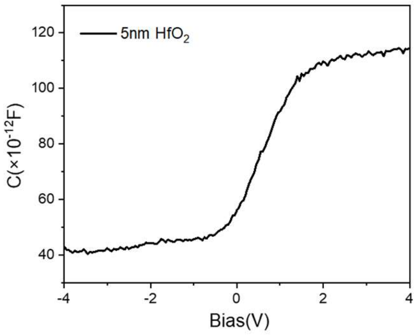

Supplementary Figure 26. The gate capacitances for the short-channel HfO_2 devices.

Supplementary Note 18. Schematic diagram and photographs of our specially designed multisource CVD setup

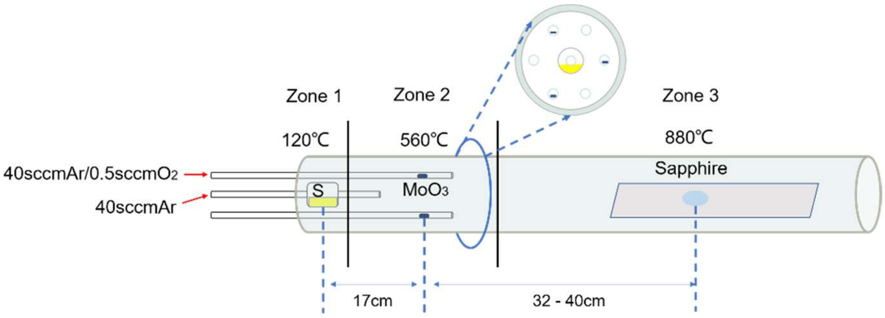

Supplementary Figure 27. Schematic diagram of the multisource CVD setup.

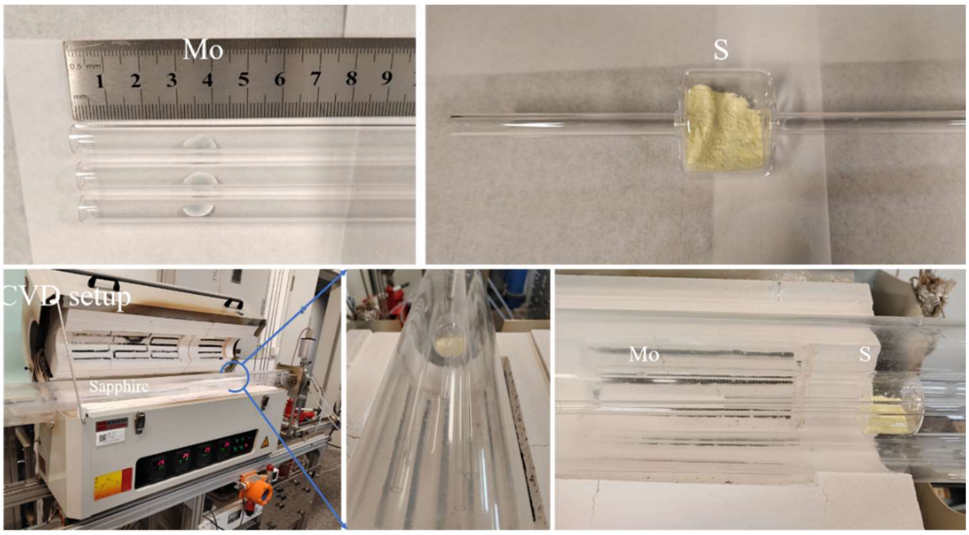

Supplementary Figure 28. Photographs of our three-temperature-zone CVD setup and the location of the solid-state sources and the substrate.

The quartz tube would develop a coating of Mo/MoO_3 after the growth, as shown in Supplementary Fig. 29. To eliminate this effect, we will anneal the used quartz tubes is placed in an air atmosphere at a high temperature (>1000°C) for 1h after the growth, and at the same time extract the exhaust gases from the high temperature annealing (residual Mo source, S source) through negative pressure pump. As can be seen from Supplementary Fig. 29, the quartz tubes after high temperature annealing are as clean as brand-new quartz tubes and will not interfere with the next growth.

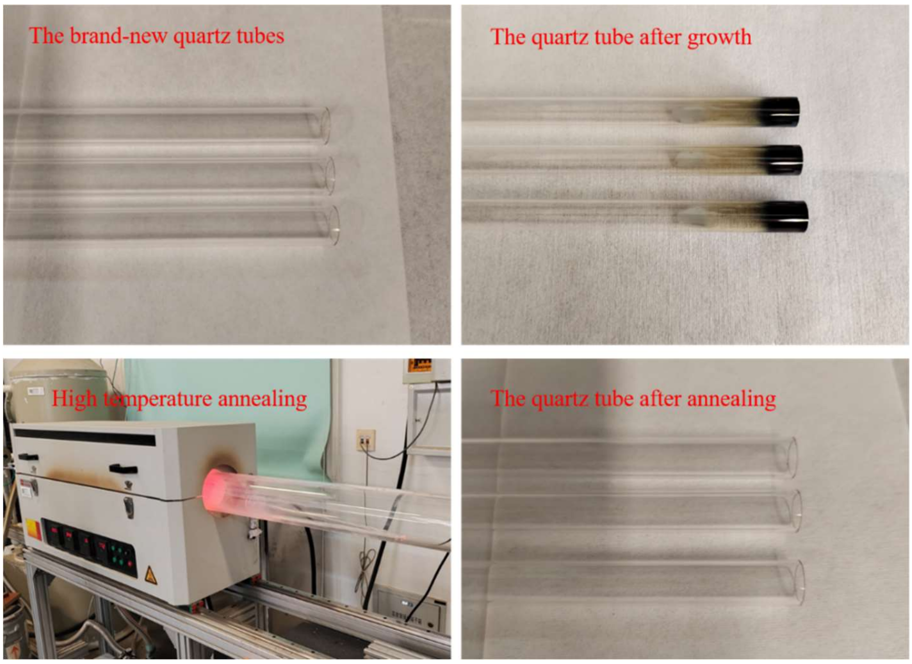

Supplementary Figure 29. The photographs of brand-new quartz tubes, the quartz tube after growth and the quartz tube after annealing (previously used one).

Supplementary Note 19. Unidirectional epitaxy at 850°C

In addition to the unidirectional epitaxy at 880°C in the main text, we also achieve the unidirectional growth at 850°C (Supplementary Fig. 30). However, at different growth temperatures, the corresponding S/MoO_3 precursor ratio is different. For example, Supplementary Figure 31 shows the degree of unidirectional alignment against the MoO_3/S precursor ratios at a growth temperature of 850°C. Clearly, the best MoO_3/S precursor ratio that show ~100% unidirectional alignment is ~2.1%. This is contrast to the best MoO_3/S precursor ratio of ~4.5% at a growth temperature of 880°C (Fig. 1c in the main text).

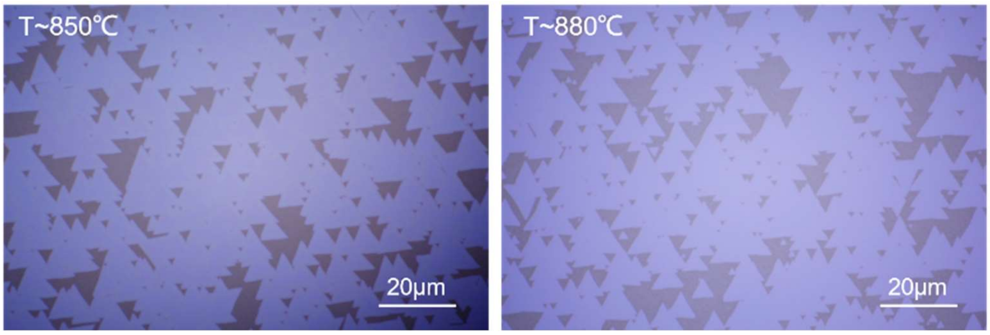

Supplementary Figure 30. Unidirectional growth of MoS_2 at different growth temperatures and different growth conditions.

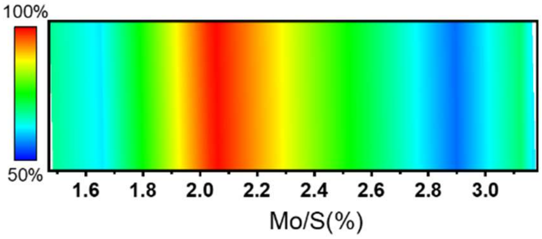

Supplementary Figure 31. Proportional changes of the single oriented MoS_2 domains grown by varying the MoO_3/S ratio at a growth temperature of 850°C.

Supplementary Note 20. General mechanism of the single-crystal TMD growth

Recently, some works have reported the growth of single-crystal TMDs on sapphire substrates[1,8-10]. However, these works rely heavily on the surface step engineering (e.g., controlling the surface step orientation and height), which typically requires the specially-designed substrates such as deliberately engineered off-cut angles or annealing at harsh temperatures. Remarkably, we realize the epitaxial growth of wafer-scale single-crystal MoS_2 monolayers on industry-compatible of general *c*-plane sapphire substrates by simply controlling the S/MoO_3 precursor ratio, showing high novelty.

Regarding the general mechanism, a universal symmetry framework has been theoretically established to guide the growth of wafer-scale single-crystal 2D materials, i.e., the symmetry group of the substrate should be a subgroup of 2D material[11]. In the light of such general guideline, *c*-plane sapphire with C_{3v} symmetry offers a possible industry-compatible substrate for the epitaxial growth of wafer-scale single-crystal MoS_2 with point group D_{3h} (C_{3v} plus a mirror-reflection symmetry σ_h). However, the adsorption energy of the most preferred domain orientation (i.e., 30°) is just slightly lower that at 90° configuration. This usually results in two antiparallel grains and twin boundaries, as in the earlier results[12,13]. Consequently, the key strategies to achieve the growth of wafer-scale single-crystal MoS_2 is to enlarge the binding energy difference between the most preferred domain orientation and its antiparallel domain. For previously reported wafer-scale growth of single-crystal TMDs via surface step engineering, it does follow the general guideline where the sapphire step edge-TMDs interaction breaks the symmetry of the antiparallel orientations. In our work, we enlarge the binding energy difference between the most preferred domain orientation and its

antiparallel domain to realize wafer-scale single-crystal MoS_2 by precisely controlling the S/MoO_3 precursor ratio.

Supplementary Table 1. Comparison of monolayer TMD electrical performance

Synthetic way	Layer	Channel length	T_{ox} (nm)	I_{on} (uA/um)	Mobility $cm^2V^{-1}s^{-1}$	On/off	Ref.
**CVD**	1L-MoS_2	200nm	30nm HfO_2	535	140	10^9	This work
**CVD**	1L-MoS_2	8.2nm	6nm HfO_2	2.5	1.1	10^6	[14]
**CVD**	1L-MoS_2	15nm	10nm HfO_2	5	\	10^6	[15]
**CVD**	1L-MoS_2	50nm	5nm HfO_2	156	21.4	10^6	[16]
**CVD**	1L-MoS_2	2μm	300nm SiO_2	18	122.6	\	[17]
**CVD**	1L-MoS_2	72nm	30nm SiO_2	320	35.7	\	[18]
**CVD**	1L-MoS_2	8.3μm	270nm SiO_2	6	43	\	[19]
**CVD**	1L-MoS_2	20μm	300nm SiO_2	0.14	7	10^6	[20]
**CVD**	1L-MoS_2	500nm	30nm Al_2O_3	450	102.6	10^9	[21]
**CVD**	1L-MoS_2	10μm	35nm Al_2O_3	6	75	10^8	[22]
**CVD**	1L-MoS_2	4μm	20nm Al_2O_3	16.7	30	10^{10}	[23]
**CVD**	1L-MoS_2	1μm	15nm HfO_2	55	54	\	[24]
**MOCVD**	1L-MoS_2	1.6μm	285nm SiO_2	10	30	10^6	[25]
**CVD**	1L-MoS_2	1μm	285nm SiO_2	49	64	10^7	[26]
**CVD**	1L-MoS_2	100nm	285nm SiO_2	62.5	13	\	[27]
**CVD**	1L-MoS_2	10nm	5-6nm Al_2O_3	426	30	\	[28]
**CVD**	1L-MoS_2	80nm	6nm Al_2O_3	20	\	10^6	[29]
**CVD**	1L-MoS_2	400nm	30nm SiO_2	280	34	10^6	[30]
**CVD**	1L-MoS_2	450nm	30nm HfO_2	65	55	\	[31]
**Exfoliated**	1L-MoS_2	10nm	300nm SiO_2	540	26.7	10^7	[32]
**Exfoliated**	1L-MoS_2	120nm	100nm SiNx	560	\	10^7	[33]
**Exfoliated**	1L-MoS_2	500nm	30nm HfO_2	320	60(250K)	10^8	[34]
**Exfoliated**	1L-MoS_2	450nm	93nm SiO_2	240	83	10^8	[35]
**Exfoliated**	1L-MoS_2	600nm	90nm SiO_2	10	13.2	10^6	[36]
**Exfoliated**	1L-MoS_2	1.3μm	30nm HfO_2	22	320	10^6	[37]
**Exfoliated**	1L-MoS_2	1.5μm	6nm HfO_2	125	16	\	[38]
**Exfoliated**	1L-MoS_2	2μm	72nm Al_2O_3	20	11	10^3	[39]
**Exfoliated**	1L-MoS_2	1.9μm	15nm h-BN	22	\	10^9	[40]
**Exfoliated**	1L-MoS_2	2μm	300nm SiO_2	5	27.6	10^6	[41]
**Exfoliated**	1L-MoS_2	1.2μm	100nm SiO_2	85	48	10^7	[42]
**Exfoliated**	1L-MoS_2	2μm	30nm Al_2O_3	450	41	10^7	[43]
**CVD**	1L-$MoSe_2$	5μm	300nm SiO_2	\	1	10^6	[44]
**CVD**	1L-$MoSe_2$	3μm	300nm SiO_2	\	30	10^4	[45]
**CVD**	1L-WS_2	30nm	5.5nm HfO_2	322	\	\	[46]
**CVD**	1L-WS_2	\	300nm SiO_2	\	50	10^7	[47]
**CVD**	1L-WS_2	\	300nm SiO_2	\	20	10^8	[48]
**CVD**	1L-WS_2	120nm	100nm SiNx	331	\	10^7	[33]
**CVD**	1L-WSe_2	6μm	300nm SiO_2	\	150(holes)	10^7	[49]
**CVD**	1L-WSe_2	\	90nm SiO_2	\	82(holes)	\	[50]
**CVD**	1L-WSe_2	1.5μm	300nm SiO_2	7.6	40	\	[51]

**Exfoliated**	1L-WS_2	\	10nm Al_2O_3/300nm SiO_2	\	83	10^8	[52]
**Exfoliated**	1L-WSe_2	1.2μm	72nm Al_2O_3	210	142	10^6	[53]
**Exfoliated**	1L-WSe_2	9.4μm	17.5 nm ZrO_2	\	250(holes)	10^6	[54]
**Exfoliated**	1L-WSe_2	2.4μm	300nm SiO_2	34	180	\	[55]

Supplementary References

1 Fu, J.-H. *et al.* Oriented lateral growth of two-dimensional materials on c-plane sapphire. *Nat. Nanotechnol.*, **18**, 1289-1294 (2023).

2 Aljarb, A. *et al.* Interfacial Reconstructed Layer Controls the Orientation of Monolayer Transition-Metal Dichalcogenides. *ACS Nano* **17**, 10010-10018 (2023).

3 Kresse, G. & Furthmüller, J. Efficient iterative schemes for ab initio total-energy calculations using a plane-wave basis set. *Phys. Rev. B* **54**, 11169-11186 (1996).

4 Blöchl, P. E. Projector augmented-wave method. *Phys. Rev. B* **50**, 17953-17979 (1994).

5 Tkatchenko, A. & Scheffler, M. Accurate Molecular Van Der Waals Interactions from Ground-State Electron Density and Free-Atom Reference Data. *Phys. Rev. Lett.* **102**, 073005 (2009).

6 Tang, J. *et al.* Vertical Integration of 2D Building Blocks for All-2D Electronics. *Adv. Electron. Mater.* **6**, 2000550 (2020).

7 Laturia, A., Van de Put, M. L. & Vandenberghe, W. G. Dielectric properties of hexagonal boron nitride and transition metal dichalcogenides: from monolayer to bulk. *npj 2D Mater. Appl.* **2**, 6 (2018).

8 Li, T. *et al.* Epitaxial growth of wafer-scale molybdenum disulfide semiconductor single crystals on sapphire. *Nat. Nanotechnol.* **16**, 1201-1207 (2021).

9 Wang, J. *et al.* Dual-coupling-guided epitaxial growth of wafer-scale single-crystal WS_2 monolayer on vicinal a-plane sapphire. *Nat. Nanotechnol.* **17**, 33-38 (2022).

10 Zhu, H. *et al.* Step engineering for nucleation and domain orientation control in WSe_2 epitaxy on c-plane sapphire. *Nat. Nanotechnol.*, **18**, 1295–1302 (2023).

11 Dong, J., Zhang, L., Dai, X. & Ding, F. The epitaxy of 2D materials growth. *Nat. Commun.* **11**, 5862 (2020).

12 Yu, H. *et al.* Wafer-Scale Growth and Transfer of Highly-Oriented Monolayer MoS_2 Continuous Films. *ACS Nano* **11**, 12001-12007 (2017).

13 Aljarb, A. *et al.* Substrate Lattice-Guided Seed Formation Controls the Orientation of 2D Transition-Metal Dichalcogenides. *ACS Nano* **11**, 9215-9222 (2017).

14 Xu, K. *et al.* Sub-10 nm Nanopattern Architecture for 2D Material Field-Effect Transistors. *Nano Lett.* **17**, 1065-1070 (2017).

15 Nourbakhsh, A. *et al.* in *2015 Symposium on VLSI Technology (VLSI Technology).* T28-T29.

16 Zhu, Y. *et al.* Monolayer Molybdenum Disulfide Transistors with Single-Atom-Thick Gates. *Nano Lett.* **18**, 3807-3813 (2018).

17 Wang, Y. *et al.* Van der Waals contacts between three-dimensional metals and two-dimensional semiconductors. *Nature* **568**, 70-74 (2019).

18 Smithe, K. K. H., English, C. D., Suryavanshi, S. V. & Pop, E. High-Field Transport and Velocity Saturation in Synthetic Monolayer MoS_2. *Nano Lett.* **18**, 4516-4522 (2018).

19 Dumcenco, D. *et al.*. Large-Area Epitaxial Monolayer MoS_2. *ACS Nano* 9, 4611 (2015).

20 Zhang, J. *et al*. Scalable Growth of High-Quality Polycrystalline MoS_2 Monolayers on SiO_2 with Tunable Grain Sizes. *ACS Nano* 8, 6024 (2014).

21 Li, T. *et al.* Epitaxial growth of wafer-scale molybdenum disulfide semiconductor single crystals on sapphire. *Nat. Nanotechnol.* **16**, 1201-1207 (2021).

22 Li, N. *et al.* Atomic Layer Deposition of Al_2O_3 Directly on 2D Materials for High-Performance Electronics. *Adv. Mater. Interfaces* **6**, 1802055 (2019).

23 Yu, L. *et al.* in *2015 IEEE International Electron Devices Meeting (IEDM).* 32.33.31-32.33.34.

24 Meng, W. *et al.* Three-dimensional monolithic micro-LED display driven by atomically thin transistor matrix. *Nat. Nanotechnol.* **16**, 1231-1236 (2021).

25 Kang, K. *et al.* High-mobility three-atom-thick semiconducting films with wafer-scale homogeneity. *Nature* **520**, 656-660 (2015).

26 Sun, L. *et al.* Concurrent Synthesis of High-Performance Monolayer Transition Metal Disulfides. *Adv. Fun. Mater.* **27**, 1605896 (2017).

27 Liu, H. *et al.* Statistical Study of Deep Submicron Dual-Gated Field-Effect Transistors on Monolayer Chemical Vapor Deposition Molybdenum Disulfide Films. *Nano Lett.* **13**, 2640-2646 (2013).

28 English, C. D., Smithe, K. K. H., Xu, R. L. & Pop, E. in *2016 IEEE International Electron Devices Meeting (IEDM).* 5.6.1-5.6.4.

29 Cao, W., Liu, W., Kang, J. & Banerjee, K. An Ultra-Short Channel Monolayer MoS2 FET Defined By the Curvature of a Thin Nanowire. *IEEE Electron Device Letters* **37**, 1497-1500 (2016).

30 McClellan, C. J., Yalon, E., Smithe, K. K. H., Suryavanshi, S. V. & Pop, E. in *2017 75th Annual Device Research Conference (DRC).* 1-2.

31 Sanne, A. *et al.* Radio Frequency Transistors and Circuits Based on CVD MoS_2. *Nano Lett.* **15**, 5039-5045 (2015).

32 Xie, L. *et al.* Graphene-Contacted Ultrashort Channel Monolayer MoS_2 Transistors. *Adv. Mater.* **29**, 1702522 (2017).

33 Shen, P.-C. *et al.* Ultralow contact resistance between semimetal and monolayer semiconductors. *Nature* **593**, 211-217 (2021).

34 Lembke, D. & Kis, A. Breakdown of High-Performance Monolayer MoS_2 Transistors. *ACS Nano* **6**, 10070 (2012).

35 Rai, A. *et al.* Air Stable Doping and Intrinsic Mobility Enhancement in Monolayer Molybdenum Disulfide by Amorphous Titanium Suboxide Encapsulation. *Nano Lett.* **15**, 4329-4336 (2015).

36 Liu, W. *et al.* in *2013 IEEE International Electron Devices Meeting.* 19.14.11-19.14.14.

37 Radisavljevic, B., Whitwick, M. B. & Kis, A. Integrated Circuits and Logic Operations Based on Single-Layer MoS_2. *ACS Nano* **5**, 9934-9938 (2011).

38 Wang, J. *et al.* Integration of High-k Oxide on MoS_2 by Using Ozone Pretreatment for High-Performance MoS_2 Top-Gated Transistor with Thickness-Dependent Carrier Scattering Investigation. *Small* **11**, 5932-5938 (2015).

39 Kang, J., Liu, W. & Banerjee, K. High-performance MoS_2 transistors with low-resistance molybdenum contacts. *Appl. Phys. Lett.* **104**, 093106 (2014).

40 Zheng, X. *et al.* Patterning metal contacts on monolayer MoS_2 with vanishing Schottky

barriers using thermal nanolithography. *Nat. Electron.* **2**, 17-25 (2019).

41 Xie, L. *et al.* A facile and efficient dry transfer technique for two-dimensional Van derWaals heterostructure. *Chin. Phys. B* **26**, 087306 (2017).

42 Kappera, R. *et al.* Phase-engineered low-resistance contacts for ultrathin MoS_2 transistors. *Nat. Mater.* **13**, 1128-1134 (2014).

43 Yu, Z. *et al.* in *2018 IEEE International Electron Devices Meeting (IEDM).* 22.24.21-22.24.24.

44 Li, J. *et al.* Chemical Vapor Deposition of 4 Inch Wafer-Scale Monolayer $MoSe_2$. *Small Science*, 2, 2200062 (2022).

45 Meng, Y. *et al.* Repairing atomic vacancies in single-layer $MoSe_2$ field-effect transistor and its defect dynamics. *npj Quantum Materials* **2**, 16 (2017).

46 Sun, Z. *et al.* Statistical Assessment of High-Performance Scaled Double-Gate Transistors from Monolayer WS_2. *ACS Nano* **16**, 14942-14950 (2022).

47 Aji, A. S., Solís-Fernández, P., Ji, H. G., Fukuda, K. & Ago, H. High Mobility WS_2 Transistors Realized by Multilayer Graphene Electrodes and Application to High Responsivity Flexible Photodetectors. *Adv. Fun. Mater.* **27**, 1703448 (2017).

48 Yun, S. J. *et al.* Synthesis of Centimeter-Scale Monolayer Tungsten Disulfide Film on Gold Foils. *ACS Nano* **9**, 5510-5519 (2015).

49 Stoeckel, M.-A. *et al.* Boosting and Balancing Electron and Hole Mobility in Single- and Bilayer WSe_2 Devices via Tailored Molecular Functionalization. *ACS Nano* **13**, 11613-11622 (2019).

50 Ji, H. G. *et al.* Chemically Tuned p- and n-Type WSe_2 Monolayers with High Carrier Mobility for Advanced Electronics. *Adv. Mater.* **31**, 1903613 (2019).

51 Wang, Y. *et al.* P-type electrical contacts for 2D transition-metal dichalcogenides. *Nature* **610**, 61-66 (2022).

52 Cui, Y. *et al.* High-Performance Monolayer WS_2 Field-Effect Transistors on High-κ Dielectrics. *Adv. Mater.* **27**, 5230-5234 (2015).

53 Liu, W. *et al.* Role of Metal Contacts in Designing High-Performance Monolayer n-Type WSe2 Field Effect Transistors. *Nano Lett.* **13**, 1983-1990 (2013).

54 Fang, H. *et al.* High-Performance Single Layered WSe_2 p-FETs with Chemically Doped Contacts. *Nano Lett.* **12**, 3788-3792 (2012).

55 Allain, A. & Kis, A. Electron and Hole Mobilities in Single-Layer WSe_2. *ACS Nano* **8**, 7180-7185 (2014).
